# Genome-Wide Development of Polymorphic Microsatellite Markers and Association Analysis of Major Agronomic Traits in Core Germplasm Resources of Tartary Buckwheat

**DOI:** 10.3389/fpls.2022.819008

**Published:** 2022-03-15

**Authors:** Siyu Hou, Xuemei Ren, Yang Yang, Donghang Wang, Wei Du, Xinfang Wang, Hongying Li, Yuanhuai Han, Longlong Liu, Zhaoxia Sun

**Affiliations:** ^1^College of Agriculture, Institute of Agricultural Bioengineering, Shanxi Agricultural University, Taigu, China; ^2^Shanxi Key Laboratory of Minor Crop Germplasm Innovation and Molecular Breeding, Taiyuan, China; ^3^Center for Agricultural Genetic Resources Research, Shanxi Agricultural University, Taiyuan, China

**Keywords:** Tartary buckwheat, genome-wide SSR marker development, association study, core germplasm, agronomic traits

## Abstract

Tartary buckwheat (TB; *Fagopyrum tataricum* Gaertn.) is an important multigrain crop and medicinal plant, but functional genomics and molecular breeding research in this species have been lacking for quite some time. Here, genome-wide screening was performed to develop simple sequence repeat (SSR) markers associated with six major agronomic traits and the rutin contents of 97 core germplasm resources. A total of 40,901 SSR loci were identified; they were uniformly distributed throughout the TB genome, with a mean distance of 11 kb between loci. Based on these loci, 8,089 pairs of SSR primers were designed, and 101 primer pairs for polymorphic SSR loci were used to genotype the 97 core germplasm resources. The polymorphic SSR loci showed high genetic variation in these core germplasm resources, with an average polymorphic information content (PIC) value of 0.48. In addition, multiple SSR markers, such as SXAU8002 [100-grain weight (HGW)] and SXAU8006 [stem diameter (SD)], were found to be associated with agronomic traits in the two environments. Finally, based on gene functional annotation and homology analysis, a candidate gene, *FtPinG0007685500*, that may affect the node number and SD of the main stem by participating in lignin synthesis was identified. This study reports the mining of genome-wide SSR loci and the development of markers in TB, which can be used for molecular characterization of the germplasm in its gene pool. In addition, the detected markers and candidate genes could be used for marker-assisted breeding and functional gene cloning in TB.

## Introduction

Tartary buckwheat (TB; *Fagopyrum tataricum* Gaertn.) is a species of *Fagopyrum* that is recognized as an important edible crop and medicinal plant due to its high abundance of flavonoids with antioxidant activity (Zhang et al., 2012b; Hou et al., 2021). TB is cultivated in diverse ecological zones worldwide, especially as a minor crop, and it is a popular food in southwestern and northern China (Tang et al., 2016). TB has higher nutritional and economic value than common buckwheat, including higher levels of vitamin B, phenolic compounds, and other antioxidants, such as rutin (Uddin et al., 2013; Zhou et al., 2015). Rutin (quercetin-3-*O*-rutinoside) is one of many well-known flavonoids with high biological activity. This compound exerts antioxidant and anti-inflammatory effects by inhibiting blood platelet aggregation, increasing capillary strength, and reducing cholesterol levels in the blood (Luthar et al., 2020). The recommended daily dose of rutin in the human diet is approximately 50 mg/day (Brunori et al., 2010). As TB is a typical functional food resource, it is necessary to analyze the genetic basis of its important agronomic and quality traits, such as plant height (PH), yield, and rutin content.

Although TB has great nutritional and medicinal values, molecular biology research on TB has long lagged behind that on other crops due to its relatively small planting area worldwide and the comparatively late completion of genome sequencing. Recently, the publication of the TB reference genome enabled the rapid identification and breeding application of important functional genes in TB (Zhang et al., 2017). These genome data also facilitate the construction of fine genetic maps based on single nucleotide polymorphisms (SNPs), simple sequence repeats (SSRs), and sequencing-based markers, and make it possible to identify agronomically important genes by map-based cloning. In recent years, many different types of molecular markers, including amplified fragment-length polymorphisms (AFLPs) (Hou et al., 2009), random amplified polymorphic DNA (RAPD) (Sharma and Jana, 2002; Zhang et al., 2012a), intersimple sequence repeats (ISSRs) (Garima et al., 2013), and SNPs (Sohn et al., 2021), have been developed and used for genetic diversity analysis in TB. Although genetic linkage maps of several different segregating populations have been established based on SSRs and other markers, the marker density of these maps was extremely sparse, which limited the detection of QTLs for important agronomic traits of TB (Du et al., 2013; Li et al., 2017). Recently, a high-density TB genetic map was constructed by using the genotyping results from restriction site-associated DNA (RAD) sequencing, and several QTLs for 1,000-grain weight and hull type were found (Shi et al., 2021). Although traditional linkage mapping based on segregating populations has been effective for mining important functional genes of TB, there have been few reports on the use of association analysis in natural populations to explore the genetic basis of important agronomic traits in TB. Hou et al. developed a total of 23 polymorphic SSR primer pairs with 49.71 effective alleles from genome survey data and revealed the genetic relationships among 64 accessions strongly associated with their geographic regions by UPMGA clustering analysis (Hou et al., 2016). Furthermore, genomic selection breeding using genome resequencing methods for developing new SNP markers would be an effective approach for buckwheat functional gene cloning and yield improvement in the future.

China is the point of origin of buckwheat and has the most abundant germplasm resources for this crop (Tsuji and Ohnishi, 2000). These abundant resources provide a valuable gene pool for the identification and utilization of buckwheat traits. In this study, six agronomic traits and the rutin contents of 97 core TB germplasm resources were evaluated over 2 years; then, the SSR loci in the genome of TB were explored in depth. Finally, the TB accessions were genotyped for 101 representative markers chosen from 40,901 SSR loci, and association studies were conducted for the abovementioned traits. This study provides more complete and accurate information for further gene cloning and marker-assisted selection (MAS) in buckwheat breeding.

## Materials and Methods

### Tartary Buckwheat Germplasm

A total of 97 buckwheat accessions, including 78 domestic varieties and 19 foreign varieties from the United States, Nepal, Bhutan, and Russia, were used in this study (Supplementary Table 1). All materials were provided by the Institute of Agricultural Bioengineering, Shanxi Agricultural University, and grown during the cropping season (March to September of 2018 and 2019) on the experimental farm of the College of Agronomy, Shanxi Agricultural University, Taigu, Shanxi, China (37°12′N, 112°28′E). Field trials were conducted in a randomized complete block design with three replicates. Each variety was planted in 3 rows, and the plant spacing was 30 cm, with a row spacing of 50 cm. The field experiment followed standard local agronomic management practices for buckwheat.

### Agronomic Trait Measurement and Rutin Content Determination

Ten plants with consistent growth were randomly selected from each variety to investigate agronomic traits at the seed filling stage (nearly 15 days after flowering). The investigated traits included plant height (PH; cm), the number of main stem nodes (MNs), and stem diameter (SD; measured with a vernier caliper, mm). At harvest, the whole plants were carefully removed from the field to measure the root length (RL). In addition, ten similarly sized plants were randomly selected, and the branches at both ends of the main stem were separated. The number of branches was recorded as the number of lateral branches (LBs). The 100-grain weight (HGW) of each variety was measured with 3 replicates in total. Plants of a similar size were selected to reduce the error in the collection of materials and the measurement of related phenotypic traits. The best linear unbiased estimator (BLUE) values of all traits over 2 years were calculated by using the R package lme4.^1^

High performance liquid chromatography (HPLC) was used to determine the rutin contents of TB seeds. The detailed steps of this procedure were as follows: TB seeds were placed into a clean mortar and ground into a powder, which was then transferred to a 2 ml centrifuge tube, and 0.15 g of powder was accurately weighed; then, 1.5 ml of methanol (chromatographic grade) was added to the centrifuge tube, which was placed in an ultrasonic water bath for 30 min (50°C). Thereafter, the tube was centrifuged for 10 min at 10,000 × *g* at 4°C, and 20 μl of the supernatant was diluted with 1 ml of methanol. Finally, 100 μl of the extract was placed in a liquid chromatography vial protected from light. The analysis conditions were as follows: the column temperature was maintained at 30°C, the mobile phase was methanol:water at 46:54, the injection volume was 5 μl, the detection wavelength was 257 nm, and the standard sample was rutin (Sigma, purity ≥ 95%). An Agilent Series 1100 liquid chromatograph (United States) and C18 column (150 mm × 4.6 mm, 5 μm; Agilent, United States) were selected.

The standard curve was drawn according to the method of Sun et al. (2020). The linear regression equation was obtained according to the standard curve, and the contents of rutin in different samples were calculated according to the following equation (Supplementary Figure 1):


Y=11.302⁢X-84.113

The univariate linear regression equation indicated a significant relative coefficient (*R*^2^ = 0.9877) between the argument *X* (peak area of rutin compound by HPLC) and dependent variable *Y* (rutin content).

### Statistical Analysis of Phenotypic Traits

Descriptive statistical analysis was performed using IBM SPSS Statistics 24,^2^ and the genotype and environment were treated as fixed and random effects, respectively. The correlation coefficients among all agronomic traits were calculated using the R package Hmisc,^3^ and the factor extraction and analysis of agronomic traits were performed using the R package Psych.^4^ In addition, clustering analysis of the natural TB population was performed using PowerMarker 3.25.^5^

### Development of Genome-Wide Simple Sequence Repeat Markers

The reference genome information for ‘‘Pinku1’’ was downloaded from the MBKBASE website,^6^ and SSR locus information was collected by MISA^7^ (Beier et al., 2017). The filtering conditions were set as follows: 10 repeats for dinucleotides, 7 repeats for trinucleotides, and 5 repeats for tetranucleotides, pentanucleotides, and hexanucleotides. According to the location information for the obtained SSR loci, primer sequences were designed in batches, and a 200 kb interval between two primers was chosen for further analysis. All primers were synthesized by Thermo Fisher Scientific (Supplementary Table 2).

### Simple Sequence Repeat Marker Detection and Association Analysis

For each accession, the healthy leaves from three plants were pooled and chosen for DNA extraction *via* the modified CTAB method (following the manufacturer’s instructions, SL2071-Coolaber, Beijing, China). The DNA from 97 TB accessions was used as a template for PCR amplification, and the primers are listed in Supplementary Table 3. PCR products were detected by 8% polyamide gel electrophoresis with silver staining. For each SSR primer pair, different combinations of bands produced by different genotypes were counted individually, e.g., 1:1, 2:2, and 3:3, so different markers produced different numbers of alleles (Supplementary Table 4). The polymorphic information content (PIC), gene diversity, and other genetic polymorphic information of the selected SSR markers were calculated using PowerMarker 3.25. In addition, genetic distances were calculated according to Nei, cluster analysis was performed *via* the neighbor-joining method, and the genetic relationship coefficients were calculated by using Spatial Pattern Analysis of Genetic Diversity (SPAGeDi)^8^ (Hardy, 2016). The population structure was analyzed using Structure 2.3.4,^9^ and the optimal K was chosen with Structure Harvester,^10^ which used the Q matrix for the association analysis. Finally, an association study of the phenotypic data and genotypic data was carried out using Tassel 5.0,^11^ and the Q matrix was used as a covariate for adjustment (Bradbury et al., 2007).

## Results

### Agronomic Characteristics and Rutin Content Evaluation

All 7 traits examined in the two environments (2018 and 2019) and BLUE values showed approximately normal distributions with low skewness (Table 1). Among the traits, HGW, LB, SD, PH, rutin, and RL showed relatively small skewness values, while MN presented a large skewness value and showed a weakly skewed distribution. The variation coefficients of the 7 traits ranged from 10.30 to 36.90%, with the minimum HGW being observed in 2018 and the maximum SD in 2019. LB, RL, and SD showed relatively large variation coefficients, indicating that the materials selected for this study were broadly representative of these traits. In general, all traits showed significant differences among genotypes and environments, and they were affected by genotype × environment interactions.

**TABLE 1:** Descriptive statistics of the main agronomic traits and rutin contents in the two environments and best linear unbiased estimator (BLUE).

Trait	Environment	Mean	SD	Range	Skewness	Coefficient of variation (%)	Genotype	Environment
100-Grain weight	2018	1.65	0.17	1.26–2.05	0.12 ± 0.24	10.30	**	**
	2019	1.79	0.25	1.18–2.58	0.28 ± 0.24	13.97		
	BLUE	1.72	0.18	1.27–2.30	0.22 ± 0.25	10.47		
Lateral branch	2018	7.60	1.54	3.33–11.00	0.03 ± 0.24	20.26	**	**
	2019	7.18	2.05	2.00–13.67	0.09 ± 0.24	28.55		
	BLUE	7.41	1.52	3.67–12.17	0.03 ± 0.25	20.51		
Root length	2018	15.23	4.04	5.70–23.23	−0.14 ± 0.24	26.53	**	**
	2019	11.64	2.77	4.00–18.67	−0.13 ± 0.24	23.79		
	BLUE	13.38	2.26	8.73–17.47	−0.27 ± 0.25	16.89		
Stem diameter	2018	8.47	1.39	5.42–13.27	0.40 ± 0.24	16.41	**	**
	2019	5.80	2.14	2.00–12.33	0.58 ± 0.24	36.90		
	BLUE	7.08	1.17	4.64–10.01	0.30 ± 0.25	16.53		
Rutin	2018	14.88	1.86	9.84–19.35	−0.42 ± 0.24	12.50	**	**
	2019	20.39	3.75	6.82–28.52	−1.67 ± 0.24	18.39		
	BLUE	17.59	2.22	9.46–22.22	−1.17 ± 0.24	12.62		
Plant height	2018	142.24	23.52	85.05–215.30	0.49 ± 0.24	16.54	**	**
	2019	130.92	19.62	82.67–172.00	−0.07 ± 0.24	14.99		
	BLUE	136.65	14.73	98.19–170.83	−0.35 ± 0.25	10.78		
Main stem nodes	2018	24.46	3.48	14.33–40.00	0.54 ± 0.24	14.23	**	**
	2019	21.61	2.99	14.67–30.00	0.28 ± 0.24	13.84		
	BLUE	23.02	2.56	15.84–29.34	0.12 ± 0.25	11.12		

**Significant at p < 0.01.

### Correlation Coefficients of Agronomic Traits and Rutin Contents

The basic correlations between different agronomic traits were calculated using Pearson’s approach in this study. SD was significantly and positively correlated with LB and PH in 2 years, with correlation coefficients of 0.37–0.60 and 0.54–0.63, respectively (Supplementary Table 5). SD and HGW showed a significant positive correlation, with a correlation coefficient of 0.36 in 2018. RL and SD and MN and SD showed significant positive correlations (0.49 and 0.69, respectively) in 2019. In addition, there was a significant negative correlation between rutin content and PH in 2019. PH was influenced by many other traits, such as SD, LB, and MN, in the two environments. In addition, the BLUE values of RL, LB, MN, SD, and PH showed significant positive correlations, as follows: RL-LB with a coefficient of 0.26, LB-MN with a coefficient of 0.26, LB-SD with a coefficient of 0.44, PH-MN with a coefficient of 0.28, PH-SD with a coefficient of 0.49, and MN-SD with a coefficient of 0.48 (Table 2). Overall, these agronomic traits showed relatively stable correlations in the two environments.

**TABLE 2:** Correlations between the BLUE values of six main agronomic traits and rutin content.

Trait	RL	LB	PH	HGW	Rutin	MN	SD
RL	1						
LB	0.26*	1					
PH	0.14	0.09	1				
HGW	0.11	−0.19	0.12	1			
Rutin	−0.07	−0.13	0.07	0.02	1		
MN	−0.12	0.26*	0.28**	−0.1	−0.16	1	
SD	−0.03	0.44**	0.49**	−0.01	−0.11	0.48**	1

*RL, Root length; LB, Number of lateral branches; PH, Plant height; HGW, 100-Grain weight; Rutin, Rutin content; MN, Number of main stem nodes; and SD, Stem diameter.*

*Significant at p < 0.05; **Significant at p < 0.01.

### Simple Sequence Repeat Markers and Genetic Structural Analysis of Tartary Buckwheat

A total of 40,901 SSR loci were uniformly distributed across the whole TB genome, with a mean distance of 11 kb between loci. There were 26,156 dinucleotide repeat SSRs, accounting for 63.95% of the total SSR loci, and the remaining SSRs were trinucleotides (28.60%), tetranucleotides (5.52%), pentanucleotides (1.36%), and hexanucleotides (0.57%) (Supplementary Table 6). The average distribution density of SSR loci on the 8 chromosomes (Chrs) ranged from 87.69 to 92.27 Mb, and the density on Chrs 4 and 8 was relatively high at 91.32 and 92.27 Mb, respectively.

**FIGURE 1:**
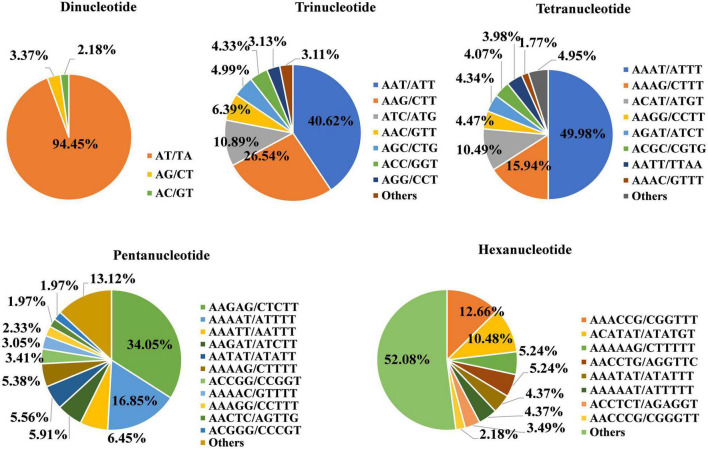
Proportions of different types of simple sequence repeat (SSR) units in the Tartary buckwheat (TB) genome. Each pie chart represents the SSR loci with different repeat units in the genome.

Among the different types of SSR markers, the identified motifs for the dinucleotide repeats included four types, namely, AT/TA, AG/CT, AC/GT, and CG/GC (Figure 1). Among these types, the greatest number (24,705) of motifs was AT/TA, accounting for 94.45% of all dinucleotide repeats, while AG/CT and AC/GT accounted for 3.37% (881) and 2.18% (569), respectively. Only one CG/GC motif was found. There were 6 types of trinucleotide repeat units, among which AAT/ATT had the maximum number of units at 4,752, accounting for 40.62% of the total. The number of AGG/CCT repeats was the least at 366, accounting for 3.13% of all SSRs. There were 8 types of tetranucleotide repeat units, among which the number of AAAT/ATTT repeats was the highest at 1,129, accounting for 49.98%. The numbers of AAAG/CTTT, ACAT/ATGT, AAGG/CCTT, AGAT/ATCT, ACGC/CGTG, AATT/TTAA, and AAAC/GTTT repeats were 360, 237, 101, 98, 92, 90, and 40, accounting for 15.94, 10.49, 4.47, 4.34, 4.074, 3.98, and 1.77%, respectively. The number of AAGAG/CTCTT pentanucleotides was the highest at 190, accounting for 34.05%, followed by AAAAT/ATTTT (94, 16.85%); AAATT/AATTT and AAGAT/ATCTT pentanucleotides were also found. AAACCG/CGGTTT hexanucleotides accounted for 12.66%. Overall, the major repeat motifs were AT/TA (60.4%), AAT/ATT (11.62%), AAG/CTT (7.59%), ATC/ATG (3.11%), AAAT/ATTT (2.76%), AG/CT (2.15%), AAC/GTT (1.83%), AGC/CTG (1.43%), AC/GT (1.39%), and ACC/GGT (1.24%) (Supplementary Table 7).

**FIGURE 2:**
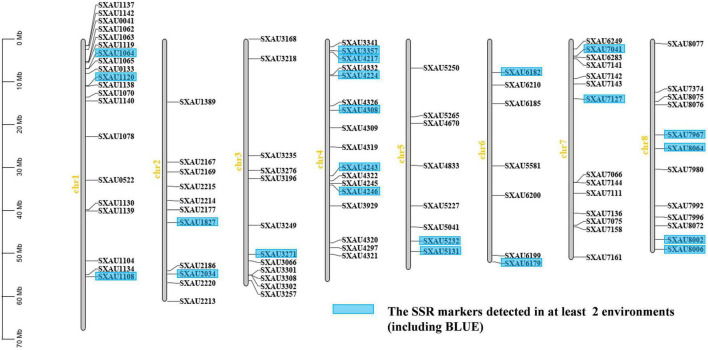
The chromosomal distribution of the 101 SSR markers used in this study.

A total of 8,089 pairs of SSR primers were designed after extracting the SSR locus sequences using the BMKCloud platform. To ensure maximum coverage of the TB genome, 1,119 of 8,089 SSR loci evenly distributed on Chrs and separated by more than 200 kb were used to detect SSR marker polymorphisms in a small natural population of 20 accessions. Finally, 101 primer pairs for polymorphic SSR loci were chosen for genotyping and association analysis (Figure 2 and Supplementary Tables 2, 3). The genome specificity of all SSR primers was verified by BLAST alignment, and all SSR primers were specific to their chromosomal locations. Here, Chr 1 had the greatest number of effective markers (20), while the fewest (7) occurred on Chr 6; the numbers of alleles for each marker varied from 2 to 7. For example, the highest number of alleles was found for the polymorphism marker SXAU3168 (Chr 3) at 7, while the average number of polymorphic loci on Chr 5 was 3.79. The variation in the PIC ranged from 0.43 to 0.56, with an average of 0.48, indicating that this population was relatively rich in genetic variation (Supplementary Table 8).

**FIGURE 3:**
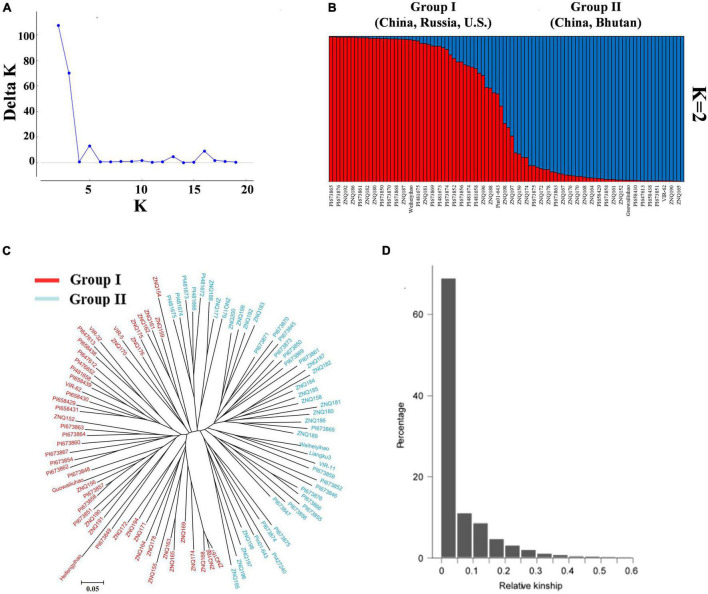
Population structure and relationship analysis of 97 core TB germplasm resources. **(A)** Distribution of Δ*K* at different *K*-values. **(B)** Population structure of 97 core TB germplasm resources inferred by genotyping 101 SSR markers (*K* = 2). Not all names of the accessions are displayed. **(C)** Neighbor-joining cluster diagram of TB resources based on SSR markers. **(D)** Affinity within the population and the proportion of different levels of kinship.

The stratification of the population structure of different genotypes can cause false positives in trait-genotype association studies. Therefore, the optimal number of subgroups (K) was calculated from the effective PIC using Structure 2.3.4, and *K* = 2 was chosen as the optimal value (Figure 3A). In addition, the population structures of the 97 germplasm resources were displayed by the Bayesian-based structure analysis with *K* = 2 (Figure 3B).

In this study, the germplasm resources were clustered *via* the “neighbor-joining” method based on Nei’s (1973) genetic distance (Figure 3C). The germplasm resources were also divided into two groups. Group I contained 49 accessions that came from Anhui (3), Guangxi (1), Gansu (4), Hubei (8), Hunan (2), Shanxi (1), Qinghai (1), and Yunnan (2) in China; the United States (8); Russia (3); Bhutan (1); and unknown locations (15). Group II contained 48 accessions that came from Guangxi (4), Guizhou (8), Sichuan (5), Hunan (1), Qinghai (1), and Yunnan (1) in China; Russia (1); Nepal (1); Bhutan (5); and unknown locations (21). The kinship values of these accessions were calculated using SPAGeDi software based on the SSR polymorphic information. The kinship value (92.71%) of the accessions was less than 0.2 (Figure 3D), indicating that the accessions exhibited low genetic relatedness at the individual level and that the population contained extensive genetic diversity.

### Association Analysis and Candidate Gene Mining

A general linear model (GLM) was used to detect the associations between SSR markers and variations in the 97 TB cultivars with thresholds of *p* < 0.05, 0.01, and 0.001. At *p* < 0.05, 144 SSR loci were significantly associated with 7 traits in 2018, among which 27, 25, 13, 13, 4, 3, and 2 SSR loci were associated with HGW, PH, MN, SD, LB, RL, and rutin content, explaining 4.3–21.35%, 5.05–18.96%, 4.23–14.84%, 4.8–24.57%, 4.16–21.0%, 4.03–15.03%, and 4.54–16.95% of the observed phenotypic variation, respectively. Sixty-seven SSR loci were significantly associated with the 7 traits in 2019. More than 10 SSR loci were associated with PH, RL, and HGW, and the explained variance was 3.5–17.0%. At *p* < 0.01, only 16 SSR loci were significantly associated with 6 traits in the two environments, with the exception of MN in 2019. At *p* < 0.001, only 1 SSR locus was significantly associated with LB and SD in the two environments, with additional loci associated with PH in 2018 and rutin content in 2019. Moreover, SXAU6182 and SXAU7127 were significantly associated with LB_BLUE.

The results of the mixed linear model (MLM) GWAS analysis showed that 55 SSR markers were significantly associated with 6 agronomic traits and rutin content at the critical significance threshold of *p* < 0.05 (Supplementary Table 9). Among them, 20, 16, and 19 SSR markers were detected for 2018, 2019, and BLUE values, respectively. Rutin content was associated with six SSRs in 2018, followed by RL (4), LB (4), SD (3), HGW (1), PH (1), and MN (1). HGW was correlated with four SSRs, followed by LB (3), SD (3), rutin content (2), MN (2), and RL (2). In addition, the BLUE values of HGW, LB, and RL were associated with 5, 4, and 4 SSR markers, respectively. Overall, relatively few SSR markers were detected for the two environments and BLUE values.

**FIGURE 4:**
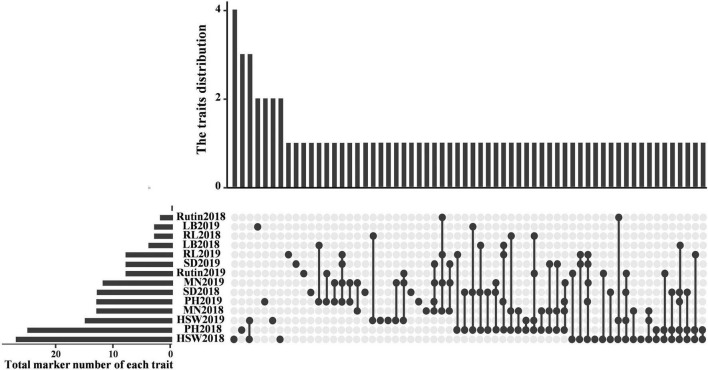
Venn diagram of associated sites based on a general linear model.

The SSR loci associated with the same trait in at least two environments were further counted and considered stable and valuable across environments (Figure 4 and Table 3). Twenty-two SSR loci were found to be associated with the same trait in at least two environments, including SXAU1108 (SD), SXAU2034 (rutin), SXAU4217 (HGW), and SXAU7127 (LB). The seven markers associated with HGW were SXAU1120, SXAU3357, SXAU4217, SXAU4308, SXAU4243, SXAU4246, and SXAU8002, respectively; the four markers associated with PH were SXAU1827, SXAU3271, SXAU5232, and SXAU8006, respectively; the three markers associated with RL were SXAU1064, SXAU4224, and SXAU8064, respectively; and the six markers associated with LB were SXAU1064, SXAU1120, SXAU6182, SXAU7127, SXAU7967, and SXAU8006, respectively. In addition, some SSR markers were significantly associated with multiple traits in both environments, such as SXAU1064 (LB and RL), SXAU1120 (SD, HGW, and LB), and SXAU8006 (SD, LB, and PH). These findings suggested that these regions could affect multiple traits in TB. Notably, SXAU4308 had significant effects on HGW in all environments at *p* < 0.05, with a phenotypic interpretation rate ranging from 0.04 to 0.17. Thus, this marker could be used as an optimal locus in marker-assisted breeding and yield improvement. In addition, SXAU4332 was significantly associated with MN in the two environments. After the detailed screening of gene functional annotations, a total of 21 candidate genes were found within the 300-kb region surrounding marker SXAU4332. Among the 21 genes, *FtPinG0007685500* encodes an MYB transcription factor containing an MYB domain, which is a homolog of the rice *MYB41* gene (LOC11429417).

**TABLE 3:** Details of the SSR markers associated with 6 agronomic traits and rutin content *via* general linear model (GLM) and mixed linear model (MLM).

SSR	Chromosome	Position (Mb)	Trait_environment^a^	Method	R^2b^
SXAU1062*	chr1	5.26	MN_2019, LB_2019*, Rutin_2019	GLM	0.09-0.14
SXAU1064*	**chr1**	**6.91**	LB_2018, LB_BLUE, RL_2019, RL_BLUE*	**GLM, MLM**	**0.03-0.06**
SXAU1065	chr1	6.99	Rutin_BLUE	GLM	0.05
SXAU1120*	**chr1**	**10.91**	SD_2018*, SD_2019*, HGW_2019*, HGW_BLUE, MN_2018, LB_2018*, LB_BLUE	**GLM, MLM**	**0.02-0.09**
SXAU0522	chr1	33.00	MN_BLUE	GLM, MLM	0.07
SXAU1130	chr1	39.90	HGW_2018, PH_2019	GLM	0.05-0.08
SXAU1108*	**chr1**	**55.45**	SD_2018, SD_2019, MN_2019*, RL_2018*	**GLM, MLM**	**0.02-0.07**
SXAU2177	chr2	39.88	LB_2019, PH_BLUE, RL_2018, Rutin_2018	GLM	0.05-0.09
**SXAU1827**	**chr2**	**42.75**	**HGW_2019, LB_2018, PH_2018, PH_BLUE, Rutin_2018**	**GLM**	**0.03-0.06**
SXAU2034*	**chr2**	**54.73**	RL_BLUE*, Rutin_2018*, Rutin_BLUE	**GLM, MLM**	**0.03-0.10**
SXAU3168	chr3	0.04	RL_2018, Rutin_2018	GLM, MLM	0.10-0.16
SXAU3249*	chr3	43.46	Rutin_2018*	GLM, MLM	0.14
SXAU3271*	**chr3**	**50.18**	MN_2019, LB_2019, PH_2019, PH_BLUE, RL_2018*	**GLM, MLM**	**0.03-0.06**
SXAU3066	chr3	51.79	Rutin_2019	GLM, MLM	0.19
SXAU3257	chr3	56.35	HGW_2019, PH_2018	GLM	0.08-0.15
**SXAU3357**	**chr4**	**2.79**	**HGW_2018, HGW_BLUE, LB_2019**	**GLM, MLM**	**0.05-0.11**
SXAU4217*	**chr4**	**3.17**	HGW_2019, HGW_BLUE*	**GLM**	**0.13-0.14**
**SXAU4224**	**chr4**	**8.61**	**RL_2018, RL_BLUE**	**GLM, MLM**	**0.07-0.09**
**SXAU4308**	**chr4**	**16.63**	**HGW_2019, HGW_BLUE, PH_2018**	**GLM, MLM**	**0.04-0.09**
**SXAU4243**	**chr4**	**31.99**	**HGW_2019, HGW_BLUE, LB_2019**	**GLM, MLM**	**0.08-0.19**
**SXAU4246**	**chr4**	**34.10**	**HGW_2019, HGW_BLUE**	**GLM, MLM**	**0.09**
SXAU3929	chr4	38.92	Rutin_2018	GLM, MLM	0.09
SXAU4670	chr5	19.70	LB_2018, Rutin_2018	GLM, MLM	0.05-0.06
SXAU4833	chr5	29.59	LB_BLUE	GLM, MLM	0.03
SXAU5232*	**chr5**	**47.16**	PH_2019*, PH_BLUE	**GLM**	**0.03-0.05**
**SXAU5131**	**chr5**	**49.55**	**MN_2019, MN_BLUE**	**GLM, MLM**	**0.04-0.05**
SXAU6182*,**	**chr6**	**7.81**	LB_2018, LB_BLUE*,**, PH_2018	**GLM, MLM**	**0.06**
SXAU6185*	chr6	15.08	HGW_2018*	GLM	0.07
SXAU5581*	chr6	29.61	LB_BLUE*	GLM, MLM	0.06
**SXAU6179**	**chr6**	**51.93**	**SD_2018, SD_2019, PH_2019, RL_2018**	**GLM**	**0.02-0.07**
SXAU6249	chr7	2.34	PH_2019	GLM	0.04
SXAU7041*	**chr7**	**4.00**	Rutin_2018*, Rutin_BLUE*	**GLM, MLM**	**0.11**
SXAU7127*,**	**chr7**	**14.03**	LB_2019, LB_BLUE*,**, MN_BLUE	**GLM, MLM**	**0.06-0.12**
SXAU7066	chr7	33.54	Rutin_BLUE	GLM	0.07
SXAU7111	chr7	35.99	Rutin_BLUE	GLM, MLM	0.1
SXAU7136	chr7	40.69	Rutin_BLUE	GLM	0.07
SXAU7075	chr7	43.41	Rutin_BLUE	GLM	0.09
**SXAU7967**	**chr8**	**22.34**	**LB_2019, LB_BLUE**	**GLM, MLM**	**0.04**
**SXAU8064**	**chr8**	**25.58**	**RL_2019, RL_BLUE**	**GLM, MLM**	**0.05-0.06**
SXAU7992	chr8	38.94	PH_2019, RL_2018	GLM	0.04-0.05
SXAU7996	chr8	41.57	LB_2019, Rutin_2018	GLM, MLM	0.07-0.10
SXAU8002*	**chr8**	**46.79**	HGW_2018, HGW_2019**, HGW_BLUE**	**GLM, MLM**	**0.04-0.17**
SXAU8006*,**	**chr8**	**48.99**	SD_2018*,**, SD_2019*,**, LB_2018*,**, LB_2019*,**, PH_2018*,**, PH_BLUE*,**, Rutin_2019*,**	**MLM**	**0.05-0.12**

*^a^The trait-environment combination of QTLs. RL, Root length; LB, Number of lateral branches; PH, Plant height; HGW, 100-Grain weight; Rutin, Rutin content; MN, Number of main stem nodes; and SD, Stem diameter.*

*^b^R^2^ represents the proportion of phenotypic variance explained by the corresponding SSR markers.*

*Bold represents the markers identified as being associated with the traits in at least two environments (including BLUE).*

*These markers were significantly associated with the trait at the p < 0.01 level.

**These markers were significantly associated with the trait at the p < 0.001 level.

## Discussion

Agronomic traits are the unique traits of crop varieties that are determined by their genetic background and the influence of the environment (Cao et al., 2016). The accurate investigation of agronomic traits contributes to the utilization of excellent traits and germplasm innovation (Jia et al., 2013). Brunori et al. (2012) found that the yield traits of 13 TB varieties from different regions were significantly different. In our study, significant differences in phenotypic characteristics and quality characteristics (rutin content) were found among different TB varieties and environments. These results indicated that these varieties show differences in adaptability to the environment and certain differences in phenotype. Therefore, it is necessary to cultivate new varieties that can adapt to complex environmental conditions and present excellent comprehensive phenotypes to meet the requirements of different regions.

Previous studies showed that there were significant correlations between PH and MSN, PH and SD, and MSN and SD (Liang et al., 2020). Similar results were obtained in this study, and SD was positively correlated with MSN and PH. In addition, PH directly affected HGW and other yield traits. Our results showed that buckwheat yield was directly affected by LB, PH, and the node number of the main stem. It was also found that PH was positively correlated with lateral branch number, HGW, and RL. Therefore, improvement of the plant-type structure is an effective direction for improving the yield and quality traits of TB.

Recently, the application of association analysis to plant molecular marker-assisted breeding has effectively accelerated the selection of superior varieties (Duan et al., 2020; Chu et al., 2021), especially in major crops. Breseghello (2006) used grain traits of 95 wheat varieties to conduct association analysis with 18 SSR markers and found that Xgwm30, Xwmc111, and Xgwm261 were significantly correlated with grain width, which contributed to related QTL studies. Agrama et al. (2007) analyzed 123 SSR markers among 92 rice varieties and obtained markers significantly associated with grain length, grain width, yield, and 1,000-grain weight. Feng et al. (2019) used GLM, MLM, and mrMLM to carry out marker-trait association analysis; 33 PSB single nucleotides were detected on 17 Chrs, and the proportion of phenotypic variation explained by the markers ranged from 1 to 32%. Lai et al. (2013) used GLMs and MLMs in the association analysis of barley SSR markers and agronomic traits and simultaneously detected 3 markers related to PH, 2 markers related to the spikelet-bearing density, and 1 marker related to awn length, with a maximum contribution rate of 6.95%.

However, the realization of association studies relies on the use of a large number of effective markers rich in polymorphic information, which have been lacking in TB studies. Based on high-throughput data, we conducted a detailed screening of the variation in SSR loci in the TB genome. Here, 8,089 SSR loci were identified in the TB genome, and 101 loci with high polymorphism were detected in 97 accessions. These SSR markers greatly enriched the TB molecular marker database, and multiple loci were confirmed to be related to important agronomic traits of TB. In this study, seven SSR markers associated with HGW, four markers associated with PH, three markers associated with RL, and six markers associated with LB were obtained by association analysis using 101 SSR loci in the two environments. Their phenotypic interpretation rates ranged from 1.32 to 5.07%, which lays a foundation for MAS in the cultivation of excellent TB varieties. In addition, an MYB transcription factor gene (*FtPinG0007685500*) was found near the significant marker SXAU4332; this gene is homologous to *MYB92* with high similarity of 97.73%. *MYB92* was confirmed to be involved in the formation of the lifetime cell wall and control lignin synthesis in hybrid poplars. The SXAU4332 marker was significantly correlated with the node number and the SD of the main stem, and *FtPinG0007685500* may have a similar function in TB. Although additional experimental verification is necessary, *FtPinG0007685500* can still be regarded as an important candidate gene affecting the node number and SD of the main stem. In general, these SSR markers and important candidate loci and genes will contribute to the improvement of important agronomic and quality traits and molecular breeding in TB.

## Data Availability Statement

The datasets presented in this study can be found in online repositories. The names of the repository/repositories and accession number(s) can be found in the article/Supplementary Material.

## Author Contributions

ZS and SH conceived and designed the experiments. SH, XR, and YY wrote the manuscript. DW, WD, and XW performed the experiments and analyzed the data. YH, LL, HL, and SH administrated the project. YH and SH revised the manuscript. All authors have read and gave final approval for publication.

## Conflict of Interest

The authors declare that the research was conducted in the absence of any commercial or financial relationships that could be construed as a potential conflict of interest.

## Publisher’s Note

All claims expressed in this article are solely those of the authors and do not necessarily represent those of their affiliated organizations, or those of the publisher, the editors and the reviewers. Any product that may be evaluated in this article, or claim that may be made by its manufacturer, is not guaranteed or endorsed by the publisher.

## Footnotes

1
https://github.com/lme4/lme4

2
https://www.ibm.com/cn-zh/products/spss-statistics

3
https://hbiostat.org/R/Hmisc/

4
https://personality-project.org/r/psych/

5
https://brcwebportal.cos.ncsu.edu/powermarker/

6
http://www.mbkbase.org/Pinku1/

7
https://webblast.ipk-gatersleben.de/misa/index.php?action=3&help=2

8
https://github.com/reedacartwright/spagedi

9
http://web.stanford.edu/group/pritchardlab/structure_software/release_versions/v2.3.4/html/structure.html

10
http://taylor0.biology.ucla.edu/structureHarvester/

11
https://tassel.bitbucket.io/
