# Supplementary figures and images for: Genome-Wide Development of Polymorphic Microsatellite Markers and Association Analysis of Major Agronomic Traits in Core Germplasm Resources of Tartary Buckwheat
Source: Front Plant Sci. 2022 Mar 15;13:819008. doi: 10.3389/fpls.2022.819008 (PMC8965444; doi:10.3389/fpls.2022.819008)

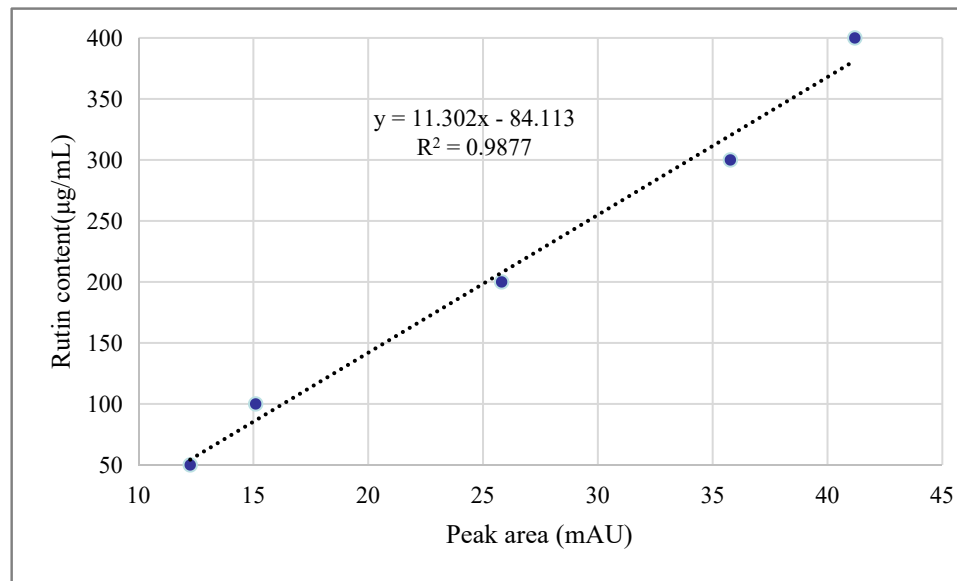

Figure S1 Standard curve of rutin content

Supplement: Supplementary file 1 [file Image_1.pdf]
